# Musculoskeletal disorder risk factors among nursing professionals in low resource settings: a cross-sectional study in Uganda

**DOI:** 10.1186/1472-6955-13-7

**Published:** 2014-02-24

**Authors:** Ian G Munabi, William Buwembo, David L Kitara, Joseph Ochieng, Erisa S Mwaka

**Affiliations:** 1 Anatomy Department, School of Biomedical Sciences, College of Health Sciences, Makerere University, P.O. Box 7072 Kampala, Uganda; 2 Surgery Department, Faculty of Medicine, Gulu University, Gulu, Uganda

**Keywords:** Musculoskeletal disorders, Risk factors, Uganda

## Abstract

**Background:**

Musculoskeletal disorders (MSD) constitute one of the main occupational hazards among health care workers. However, few epidemiological studies on work related MSD among nursing professionals have been carried out in Africa. The purpose of this study was to assess the work related musculoskeletal disorders and associated risk factors among nursing professionals in Uganda.

**Methods:**

This was a cross-sectional study of MSD among 880 nursing professionals from five selected hospitals in Uganda. Data was collected using a questionnaire adapted from the Dutch Musculoskeletal and Nordic Musculoskeletal questionnaires. Descriptive (mean, standard deviation and percentages) and inferential (Chi square test and logistic regression analysis) statistics were used to analyse data. Alpha level was set at p < 0.05.

**Results:**

A total of 741 completed questionnaires were analysed (response rate 85.4%). The average age of the respondents was 35.4 (SD 10.7) years and a majority were female (85.7%). The average working hours per week was 43.7 (SD 18.9 hours). The 12-month period-prevalence of MSD at anybody site was 80.8%. The most common site of MSD was the lower back (61.9%). Significant risk factors for reported MSD included often working in a slightly bent posture (adjOR 2.25, 95% CI 1.20-4.26), often working in a slightly twisted posture for long (adjOR 1.97, 95% CI 1.03-3.77), mental exhaustion (adjOR 2.05, 95% CI 1.17-3.5), being absent from the work station for more than 6 months due to illness or an accident (adjO|R, 4.35, 95% CI 1.44-13.08) and feeling rested after a break (adjOR 2.09, 95% CI 1.16-3.76).

**Conclusions:**

Musculoskeletal disorders affect more than 80% of nursing professionals in Uganda with the most commonly, affected site being the lower back. Significant risk factors for MSD include; being absent from the work station for more than 6 months due to illness or an accident, working in awkward postures, pushing/pulling of heavy loads and mental exhaustion. There is a need for greater advocacy, better working conditions and adoption of strategies to reduce occupational injuries.

## Background

Africa, with 24 percent of the global burden of disease, employs only three percent of all health professionals [1]. Nurses in Africa are arguably the most important health care workers available in most sub-Saharan nations, performing a broad range of tasks and working in settings where no other health workers, including physicians, are available [2]. In Uganda the acute shortage of nursing professionals stands at a dismal six nurses to 100,000 populations [3], with the result that the available few have to work under relatively difficult conditions.

Musculoskeletal disorders (MSD) have been described as one of the main occupational hazards among frontline health care workers in whom they present as a major occupational problem and a significant cause of morbidity [4]. Nursing professionals are commonly identified as being at risk for patient handling injuries, but many other health care professionals providing direct care during the course of a patient’s hospital stay are also potentially at risk. Patient handling has been identified as a significant contributor to musculoskeletal injuries among nurses and nurses’ aides especially injuries to the back, neck, and shoulders. Despite their large demographic and associated potential for occupational health problems, few epidemiological studies have investigated MSD risk factors among African nursing professionals [5,6]. The main purpose of this study was to assess the work related musculoskeletal disorders, and associated risk factors among nursing professionals in Uganda.

## Methods

### Study design and setting

This was a cross-sectional study of nursing professionals MSD from 5 selected hospitals in Uganda. The hospitals included a purposive sample of 3 regional referral hospitals, one non-governmental private not for profit hospital and one private for profit hospital. The sample size was calculated using the online OpenEpi software for sample size calculation for proportions (http://www.openepi.com) [7]. We used an assumed prevalence of MSD of 78% as was reported in the study by Tinubu et al. [6] and precision of 5% (delta). To allow for adequate power during sub analysis a confidence level of 99.9% for (1-β) was selected to give a sample size of 743 nursing professionals. To this we included a 20% additional allowance for non- response. This gave a final targeted sample size of 890 nursing professionals. All nursing professionals in the hospitals were eligible for inclusion in the study.

### Questionnaire design

Data was gathered by means of an anonymous, self-reporting questionnaire which was adapted from the standardized Dutch Musculoskeletal (DMQ) and Nordic Musculoskeletal questionnaires (NMQ) [8,9]. These questionnaires have been used extensively in research studies on MSD in the general population and among different occupational groups [10-16]. The NMQ is a useful tool for surveillance of work related MSD with an excellent sensitivity (82.3%- 100%), specificity (51.1%- 100%) and a high repeatability (Kappa = 0.63-0.90). The DMQ has been shown to have good reliability (Cronbach’s alpha >0.80) [9,17-21]. Cross cultural adaptation and psychometric evaluation of the NMQ and physical work related items of the DMQ have also been done [22,23]. The DMQ is a validated tool for the analysis of musculoskeletal workload and associated potential hazardous working conditions as well as musculoskeletal symptoms in worker populations. The DMQ enables a global assessment of musculoskeletal workload and other potentially hazardous working conditions by seven homogeneous indices (forces, dynamic loads, static loads, repetitive loads, climatic factors, vibration and ergonomic environmental factors) and four separate factors (sitting, standing, walking, uncomfortable postures).

The questionnaire was pre-tested, simplified and adjusted to the Ugandan setting for easy understanding. It comprised of a simple tick-box format, with the first section covering demographic (individual) items such as age, tobacco smoking, alcohol consumption, marital status, and number of children. The second section focused on workplace factors such as work hours, shift work, and career duration as a nursing professional. Questions addressing psychosocial factors constituted the third component, and were adapted from the DMQ [8]. Further items included their daily physical tasks, posture, and perceived intensity of work.

MSD were classified according to the criteria of Kuorinka et al. [9], who defined them as an ache, pain discomfort or numbness in the defined area over a set period of time. MSD questions included an anatomical diagram with specifically shaded areas, which focused on the occurrence of symptoms at certain body sites over the previous 12-month period and whether sick leave had been taken or medical advice sought [9]. A 12-month recall period was used for MSD, as this has been shown to be an appropriate time-scale in other studies [10].

### Ethical considerations

Permission of the administrators of the selected research hospitals was obtained before the commencement of the study. The study was conducted in accordance with three basic ethical principles, namely respect for persons, beneficence and justice. Ethical review and approval was sought from the Makerere University School of Biomedical Sciences Research and Ethics Committee. Written informed consent for participation in the study was obtained from all participants. Questionnaires were distributed to the 5 hospitals and an ample time of 2 weeks was given for their completion.

### Data analyses

Data was entered into Epidata version 3.2 (Epidata association, Denmark) and exported to STATA 12 (StataCorp LP, Texas, USA) where after checking for duplicate entries a preliminary analysis of each variable was made to identify additional range and omission errors. Once the data was cleaned the results of the analysis were then summarized using frequencies and descriptive statistics to summarize the data. Subsequent analysis was made with various tests including multiple response analysis to describe the MSD and its associated risk factors for the study population. Chi-square cross tabulations were used to distinguish differences in reported MSD among the different nursing cadres. Data was stratified by MSD subcategory (neck, shoulder, elbows, upper back, wrist/hands, lower back, knees, ankles/feet and any MSD. MSD was used as the dependent variable, with demographic (individual) factors, workplace factors, and psychosocial factors used as the independent variables. Univariate logistic regression analysis was used to identify a few key risk factors for this study population followed by multivariable logistic regression of only the significant items to create a single multifactorial model of MSD for the study population. Risk factors were calculated using logistic regression later presented as Odds Ratios (OR) and Adjusted Odds ratios (adjOR) with 95% Confidence Intervals (95% CI). The level of significance was set at <0.05.

## Results

### Demographic factors and prevalence

A total of 880 questionnaires were distributed of which 755 completed the questionnaire giving a response rate of 85.4%. These 755 nursing professionals participated keeping the power (1-β) of the study above the calculated 99.9% for the targeted 743 nursing professionals.

Respondents in the study comprised of nurses (58.4%), midwives (28.9%), theatre staff (3.8%) and nurse aides (8.9%). The average age was 35.4 (SD 10.7) years, range 18–62 years) and the majority were female (85.7%). About 59.5% were married. The average working hours per week was 43.7 (SD 18.9) hours and the mean career duration of 11.9 (SD10.5) years.

In this study 598 of 741 respondents reported having experienced an ache, pain or numbness in any one region of their bodies in the 12 months prior to the study giving a self-reported 12 month MSD at anybody site prevalence of 80.8% (Table 1). Overall the most common sites of reported MSD were; the lower back (61.9%), feet and ankles (38.1%), knees (37.1%), neck (36.9%), upper back (35.8%), and the shoulders (32.6%).

**Table 1:** Pattern of musculoskeletal disorders among nursing professionals

**MSD**	**Frequency**	**Percentage**
Neck	226	36.9
Shoulders	231	32.6
Upper back	256	35.8
Elbows	109	15.4
Wrists	206	29.1
Lower back	445	61.9
Hips	198	27.9
Knees	267	37.1
Ankle and feet	275	38.1
**Any MSD**	**596**	**80.8**

### MSD risk factors

Additional file 1: Table S2 shows the association between the 12 month self-reported MSD in the different body regions and the various risk factors. The risk factors were categorized as demographic/individual factors, work place factors and psychosocial factors. Some risk factors like being married, having children, weekly working hours, work often hindered by the absence of others and manual handling of patients were excluded from the table because they were not statistically significant.

Among the individual factors age (OR 1.05, 95% CI 1.03 to 1.07), sex (OR 0.47, 95% CI 0.30 to 0.75), being married (OR 1.69, 95% CI 1.17 to 2.44) and having children (OR 1.16, 95% CI 1.02 to 1.31) had significantly increasing odds in respondents with reported MSD. For the specific sites note that female respondents were 2.26 times more likely to report lower back pain than their male counterparts (OR 2.26 95% CI 1.42 to 3.60). Midwives were most likely to have reported pain in their elbows (27.8%) compared with nurses (14.4%), nurse aides (10.2%) and theatre assistants 17.7%). This was significant (Chi square = 16.05, p value = 0.01). This pattern of significant reported pain according to occupation was also observed with the ankles/ feet (midwives, 54.6%; Nurses, 44.9%; theatre assistants, 35.3%; nurse aides, 26.5%; Chi-square value = 13.60 p value = 0.03). There were no other significant differences in reported pain for the other parts of the body according to the respondents’ occupation in the 12 months prior to the study. On considering the different anatomical regions (Table 1), the lower back was most frequently affected in 61.9% of respondents.

Among the work place factors career duration (OR 1.05, 95% CI 1.03 to 1.08), pushing/pulling loads greater than 20 kg (OR 1.47, 95% CI 1.01 to 2.13) and, working in bent and twisted postures (OR 2.02, 1.23 to 3.30) were significantly associated with MSD. Manual handling of patients was reported by 84.4% of respondents but there was no significant association with MSD (OR 1.30 95% CI 0.79 to 2.12). Several psychosocial factors were considered, those with a significant association with any MSD included; mental exhaustion (OR 2.22, 95% CI 1.51 to 3.27), nurses who supervised others (OR 1.81, 95% CI 1.23 to 2.67) and those who had been absent from their work stations in the last six months because of an illness or an accident (OR 5.91, 95% CI 2.36 to 14.79). On average respondents had 1.5 (SD 1.3 breaks) during the course of their working day, however sixty-nine percent of them reported that their breaks were insufficient (OR 0.46, 95% CI 0.32 to 0.68) and they were not rested after the breaks and this significantly contributed to their MSD (OR 0.85, 95% CI 0.58 to 1.25). About 48.7% of nurses reported that circumstances in their private lives significantly affected their work (OR 1.38, 95% CI 0.95 to 2.00). Table 2 summarises the model fitting for all the variables used in the study note that only; alcohol consumption (adjOR 0.48, 95% CI 0.26-0.87), often working in a slightly bent posture (adjOR 2.25, 95% CI 1.20-4.26), often working in a heavily bent posture (adjOR 0.43, 95% CI 0.20-0.96), working in a slightly twisted posture for long (adjOR 1.97, 95% CI 1.03-3.77), mental exhaustion (adjOR 2.05, 95% CI 1.17-3.5), being absent from the work station for more than 6 months (adjO|R, 4.35, 95% CI 1.44-13.08) and feeling rested after a break (adjOR 2.09, 95% CI 1.16-3.76) remained significant.

**Table 2:** A multivariable logistic regression analysis of the various risk factors for reported MSD

**Factor**	**Odds ratio**	**AdjOR**	**95% CI AdjOR**
Age	1.05	1.00	0.94-1.08
Sex	0.47	0.71	0.35-1.43
Married	1.69	1.24	0.70-2.17
Number of children	1.16	1.13	0.93-1.37
**Alcohol consumption**	**0.82**	**0.48**	**0.26-0.87**
**Smoking**	**0.54**	**0.06**	**0.01-0.53**
Number or hours worked per week	0.99	1.00	0.98-1.01
Career duration	1.05	1.03	0.96-1.11
Pushing/pulling loads > 20 kg	1.47	1.31	0.75-2.28
**Often work slightly bent posture**	**2.28**	**2.25**	**1.20-4.26**
**Often work in heavily bent posture**	**1.34**	**0.43**	**0.20-0.96**
**Slightly twisted posture for long**	**2.07**	**1.97**	**1.03-3.77**
Bent and twisted posture	2.02	1.73	0.75-4.02
**Mental exhaustion**	**2.22**	**2.05**	**1.17-3.58**
Work hindered by the absence of others	1.20	0.71	0.47-1.28
Supervision of other	1.81	1.36	0.77-2.41
Having part time jobs	0.93	0.50	0.22-1.16
**Being absent for more than 6 months**	**5.91**	**4.35**	**1.44-13.08**
Normal breaks sufficient	0.46	0.66	0.36-1.21
**Feeling rested after the breaks**	**0.85**	**2.09**	**1.16-3.76**
Work conditions affect private life	1.38	1.27	0.74-2.21
Manually handling patients	1.29	1.12	0.52-2.42

Items in bold remained significant after adjustment (p < 0.05).

## Discussion

This study assessed the work related musculoskeletal disorders, and associated risk factors among nursing professionals in Uganda. The study found several significant factors associated with MSD among nursing staff. These included alcohol consumption, pushing/pulling heavy loads, working in awkward postures, mental exhaustion being absent from one’s work station for more than 6 months and feeling rested after a break.

Despite their large demographic and associated potential for work related health problems, few epidemiological studies have investigated MSD risk factors among African nursing professionals. The prevalence of MSD recorded in this survey cannot be compared directly with those found in most other studies because of differences in the method of investigation and case definition of the populations studied. In our study MSD were classified according to the criteria of Kuorinka et al. [9] who designed the NMQ as were several studies among Swedish [24], Japanese [25] and Chinese nurses [26]. The self-reported 12-month period-prevalence of MSD at anybody site during our study was 80.8%, which is comparative to previous investigations of Nigerian nurses (78%) [6], Swedish nurses (84%) [24] and Japanese nurses (85.5%) [25] but higher than a Chinese investigation (70%) [26]. By individual body site, 61.9% of the respondents in our study, reported lower back as the most frequent site for MSD. This figure is much higher than the 20% prevalence of low back pain in the Ugandan population [27]. Low Back Pain (LBP) is a regular occupational problem for nurses worldwide, and has been previously reported at rates between 45% in England [28], 63% in Australia [29] and 64% in Sweden [24]. Research from Hong Kong and China has also shown that LBP may affect between 40.6% [30] and 56% [26] respectively. African studies report LBP rates between 44.1% and 79.4% [5,6]. Neck MSD rate was 36.9% which was almost similar to a United States study (35.1%) [31], but lower than those reported among European and Asian nurses with rates between 40% and 71.6% respectively [24-26,29,32]. We came across only one African study which reported a neck MSD rate of 28% [6] however the sample size was relatively small (128 respondents). Though not widely reported the knees and ankle/feet MSD were also reported but at rates relatively higher than those reported elsewhere (22% and 10.2% respectively) [6]. To the knowledge of the authors there is an apparent dearth of information on musculoskeletal pain in the Ugandan general population however community studies done elsewhere reported pain in the back (23%), knees (19%) and shoulders (16%) [33]. The lifetime prevalence of back pain is 58–84% and the point prevalence (proportion of population studied that are suffering back pain at a particular point of time) is 4–33% [34,35]. Pain and sensory disturbance in the upper limb are common symptoms in the general population with reported point prevalence rates ranging from 4% to 35% [36,37].

It can be deduced from the above prevalence rates that MSD among Ugandan nursing professionals are higher than in the general population at rates comparable to other parts of the world.

### MSD risk factors

For this population the study found that; age, gender, being married and having children had significant different odds for respondents with MSD when compared with those without MSD. Females were twice as likely to report MSDs in several regions of the body especially the lower back and lower limbs compared to their male counter parts. Many studies indicate that women have a higher musculoskeletal morbidity than men [38-40]. This has been found in studies of the general population as well as in different occupational groups. The reasons for these gender differences are not always obvious. Prevailing explanations of women’s excess health risk revolve around two basic propositions. Greater prevalence or severity of symptoms may be due to the higher demands and constraints that women face, or because women are more affected by, or vulnerable to, the health impact of particular demands and constraints [41]. In the African society women do the bulk of house-hold chores, MSDs may be a reflection of the accumulation of difference in exposures at work and at home, providing an opportunity to tease out the relationships between work-related factors, domestic load and underlying biological differences. Thus the difference between women and men in exposure, at work and at home, to risk factors for musculoskeletal disorders is one model that may explain the markedly higher prevalence of these disorders in women compared to men [38].

Alcohol consumption was found to have a protective effect on reported MSD (Table 2). Wright et al. [42] also found that people who consumed either moderate or excess quantities of alcohol reported having consulted a practitioner for back pain less often than those who did not drink at all. This might be surprising however alcohol has been associated with decreased prevalence of chronic widespread pain [43]. Studies have shown individuals often use alcohol to cope with stress [44] and chronic pain can be a significant stressor [45], others use it for pain management [45].

Three work place risk factors of MSD were identified in this study. Career duration, pushing/pulling loads greater than 20 kg (OR 1.47), often working in bent (OR 2.28) and twisted postures (OR 2.02) for long periods. Of these three, working for long periods in a slightly bent position remained significant after adjusting for all the other variables. This is not surprising because most of the hospitals in Uganda are poorly equipped and understaffed [46]. Most hospitals do not have patient lifting equipment therefore patients have to either be lifted or be pushed on mal-functioning trolleys to and from theatre, emergency and in between other hospital departments.

Pushing and pulling of heavy load in our study mostly affected the neck, elbows and the lower back. This differed with Smedley et al. [47] who observed that pushing/pulling seemed to be harder on subjects’ shoulders than on their backs.

Musculoskeletal pain among hospital nurses has also been associated with some actual tasks and items related to work postures, work control, and work organization [48]. Several studies have implicated manual handling of patients’ physical loads as predictors of MSDs and low back pain among nurses [10,28-30,49,50] however in this study there was no significant association between MSDs and manual handling of patients. People with MSD symptoms may have been selected out of the most physically demanding jobs. Some hospitals in Uganda have a tendency of allocating lighter duties to nursing professionals with co morbidities especially the elderly, those with physical disabilities and those with chronic pain. This therefore shields them from patient handling activities and would tend to obscure associations between MSD and patient handling activities. Because of the potential for such biases, the findings require further testing with a prospective study design where activities are ascertained in people who are pain free, and they are then followed up to assess the subsequent incidence of symptoms.

Among the nursing professionals surveyed, midwives were most likely to develop MSDs especially at the elbows and the ankles. Uganda has one of the highest fertility rates in the world however it has numerous maternal and child health challenges [51]. Midwives work in appalling conditions many hospitals have poorly equipped and badly designed labour suites with mainly low lying delivery beds [52]. After a full, busy workday, the physiological patterns of labour and birth and the desirability of continuity of care may dictate that the workday for midwives extend far into the night with fatigue, sleep deprivation and the potential for work-family conflict [52] adding to the pressures. Working under these conditions may result in injury [53] and subsequent workforce attrition [54]. Some studies have shown that physical and psychological demands might cause health care workers to leave their profession [55,56]. In a survey of over 43,000 nurses in five countries, 17% to 39% reported that they planned to leave their job in the next year due to the physical and psychological demands of the profession [57]. These findings are especially disturbing given the current shortage of nurses and the increasing need for nursing care projected over the next decade [58].

Psychosocial factors have been identified as strong risks of MSD [26,59,60]. Mental exhaustion was found to be associated with an almost 2 fold increase in reported MSD in this study (see Table 2). A previous study by Nabirye et al. [46] reported that Ugandan nurses had high occupational stress levels especially the older age group and those working in public hospitals, and this significantly affected their performance at work. This is also consistent with studies done among Asian nurses who reported high mental pressure as a significant risk factor for MSD [25,26]. Supervision of others, circumstances in private life and absenteeism from work due to illness were also identified as risk factors for MSD (Additional file 1: Table S2). There are several push factors which significantly affect the productivity of health workers and these include; poor remuneration and conditions of service, civil unrest lack of opportunities for postgraduate training, feelings of lack of respect/value placed in health workers by country/system, and concern about poor governance and management of the health system [61,62]. Feeling rested after a break off work had a significant association with reported MSD. This might be due to the fact that when one is experiencing pain in the body, a period of rest will reduce the pain and he/she will feel some relief whereas if one initially had no pain then the difference might not be as obvious.

Interpretation of our study results must take into account several limitations of the study design. Errors may have occurred from biased recall of symptoms or activities and hospitals did not have a uniform number of nursing staff.

## Conclusions

Work related MSD are relatively common among nursing professionals in Uganda. The etiology of MSD among nursing professionals is multi-factorial. Among the individual factors age, gender, being married and having children had significant association with MSD. Significant work place risk factors included the pushing/pulling of heavy loads and working in awkward postures. Mental exhaustion, supervision of others, circumstances in private life and absenteeism from work due to illness were the psychosocial factors identified as risk factors for MSD. To help alleviate the considerable MSD burden, there should be greater advocacy, increased recruitment of more human resources, better working conditions, reduction of the work overload and increase job satisfaction and job performance, and adoption of strategies to reduce manual patient handling activities.

### Implication for nursing practice

The results of this study have implications for nursing practice. Most hospitals in Uganda are poorly equipped, have low staffing levels and the health workers are poorly remunerated. This leads nursing professionals to seek for part-time jobs, resulting in long working hours (average weekly work hours for this study was 43.7 hours) and poor job performance. Work-related musculoskeletal injuries negatively impact employees’ quality of life, patient care, and health care facilities’ profit margin. The consequences of musculoskeletal injuries have included job change, job loss, and chronic pain [63]. Nurses in low-income countries are migrating to wealthier countries in search of better salaries, improved working conditions, and more opportunities for further training, resulting in a “brain drain” [64]. A study done among nursing students at 2 Ugandan universities on factors that would influence their future practice locations revealed that 70% had the intent to migrate abroad within the next 5 years with dissatisfaction with financial remuneration being their main reason [65]. Therefore to retain the few nurses we have in the country, there is a need to improve on their working conditions and to come up with effective early prevention strategies against work related MSD.

### Implication for management

Occupational health is a field that has been neglected in Uganda, there is no forum for addressing work related musculoskeletal injuries especially in public hospitals. The results of this study challenge the ministry of health to create a culture of safety for health care workers, which will improve on their performance and patient safety. The Ugandan health system needs to create a culture that encourages injury and potential safety violation reporting so incidents can be prevented. Hospitals too should create a culture of safety whereby everyone in the facility is looking for ways to decrease injuries and improve safety. Nurses should report potential risks, make sure that all serious injuries are reported, and occupational health teams are formed in hospitals to improve safety. Nursing training curricula must address safe body mechanics, injury prevention, and safety improvement.

## Abbreviations

MSD: Musculoskeletal injuries and disorders; LBP: Low back pain.

## Competing interests

The authors declare no conflict of interest.

## Authors’ contributions

ESM and IGM conceived and designed the study. All contributed in the data collection. ESM, IGM and WB analyzed the data. JO, DLK and ESM prepared the manuscript, final proof reading and approval of the manuscript was done by OJ. All authors read and approved the final manuscript.

## Pre-publication history

The pre-publication history for this paper can be accessed here:

http://www.biomedcentral.com/1472-6955/13/7/prepub

## Supplementary Material

Additional file 1: Table S2 The association between the 12 month Self-reported MSD in the different body regions and the various risk factors. Click here for file
