# Supplementary material for: Musculoskeletal disorder risk factors among nursing professionals in low resource settings: a cross-sectional study in Uganda
Source: BMC Nurs. 2014 Feb 24;13:7. doi: 10.1186/1472-6955-13-7 (PMC3940025; doi:10.1186/1472-6955-13-7)
Supplement: Additional file 1: Table S2 — The association between the 12 month Self-reported MSD in the different body regions and the various risk factors. [file 1472-6955-13-7-S1.doc]

Table2: The association between the 12 month self reported MSD in the different body regions and the various risk factors.

| Risk factors^a^ | Neck | | Shoulders | | Upper back | | Elbows | | Wrist/hand | |
| --- | --- | --- | --- | --- | --- | --- | --- | --- | --- | --- |
|  | OR | 95% CI | OR | 95% CI | OR | 95% CI | OR | 95% CI | OR | 95% CI |
| **Demographic (Individual) factors** |  |  |  |  |  |  |  |  |  |  |
| Sex | 1.07 | 0.7-1.65 | 0.77 | 0.48-1.24 | 1.04 | 0.67-1.61 | 0.80 | 0.43 -1.5 | **0.53** | **0.32-0.91^*^** |
| Age | 0.99 | 0.98-1.01 | 1.00 | 0.99-1.02 | 1.01 | 1.01-1.03 | 1.01 | 0.99-1.03 | **1.02** | **1.01-1.04^*^** |
| **Work place factors** |  |  |  |  |  |  |  |  |  |  |
| Career duration | 1.00 | 0.99-1.02 | 1.01 | 1.00-1.03 | **1.02** | **1.00-1.03^*^** | 1.03 | 1.00-1.04 | **1.03** | **1.01-1.04^*^** |
| Pushing/pulling loads > 20kg | **1.41** | **1.03-1.9^*^** | 1.15 | 0.83-1.59 | 1.09 | 0.80-1.49 | **1.63** | **1.05-2.53^*^** | 1.29 | 0.92-1.81 |
| often work slightly bent posture | **1.76** | **1.16 -2.66^*^** | **1.57** | **1.03-2.4^*^** | 1.43 | 0.95-2.15 | 1.22 | 0.71-2.10 | **2.27** | **1.40-3.69^*^** |
| often work in heavily bent posture | 1.35 | 0.98 -1.85 | 1.23 | 0.88-1.71 | 1.19 | 0.87-1.64 | **1.93** | **1.28-2.93^*^** | **1.62** | **1.15-2.26^*^** |
| slightly twisted posture for long | **1.68** | **1.22 -2.31^*^** | **1.79** | **1.28-2.48^*^** | 1.37 | 0.99-1.89 | 1.14 | 0.74 -1.74 | **1.41** | **1.01-1.98^*^** |
| Bent and twisted posture | **1.84** | **1.3-2.6^*^** | **1.72** | **1.2-2.47^*^** | 1.37 | 0.96-1.95 | 1.58 | 1.01 -2.48 | **1.62** | **1.12-2.34^*^** |
| **Psychosocial factors** |  |  |  |  |  |  |  |  |  |  |
| Mental exhaustion | **1.53** | **1.12-2.08^*^** | **2.14** | **1.54-2.98^*^** | **1.42** | **1.04-1.95^*^** | **2.02** | **1.30-3.13** | **1.91** | **1.36-2.68^*^** |
| Having part time jobs | 1.11 | 0.68-1.82 | 0.68 | 0.4-1.18 | 0.75 | 0.44-1.26 | 0.95 | 0.482-1.87 | **0.49** | **0.26-0.91^*^** |
| Supervision of others | 1.08 | 0.77-1.52 | 1.19 | 0.83-1.7 | **1.53** | **1.07-2.19^*^** | **1.80** | **1.07-3.02^*^** | 1.39 | 0.96-2.04 |

| **Risk factors^a^** | **Lower back** | | **Hips** | | **Knees** | | **Ankles and feet** | | **Total MSD** | |
| --- | --- | --- | --- | --- | --- | --- | --- | --- | --- | --- |
|  | OR | 95% CI | OR | 95% CI | OR | 95% CI | OR | 95% CI | OR | 95% CI |
| **Demographic (individual) factors** |  |  |  |  |  |  |  |  |  |  |
| Sex | **0.46** | **0.3-0.70^*^** | **0.31** | **0.17-0.59^*^** | **0.40** | **0.24-0.67^*^** | **0.46** | **0.28-0.75^*^** | **0.47** | **0.30-0.75^*^** |
| Age | **1.04** | **1.02-1.05^*^** | **1.04** | **1.02-1.06^*^** | **1.05** | **1.04-1.07^*^** | **1.07** | **1.05-1.09^*^** | **1.05** | **1.03-1.07^*^** |
| Married | **1.80** | **1.32 -2.43^*^** | 1.29 | 0.91 -1.82 | **1.51** | **1.1-2.07^*^** | **1.40** | **1.02-1.92^*^** | **1.69** | **1.17-2.44^*^** |
| number of children | 1.08 | 0.99-1.12 | 1.09 | 0.99-1.19 | **1.13** | **1.04-1.23^*^** | 1.05 | 0.97-1.14 | **1.16** | **1.02-1.31^*^** |
| **Work place factors** |  |  |  |  |  |  |  |  |  |  |
| Career duration | **1.04** | **1.02-1.05^*^** | **1.04** | **1.02-1.06^*^** | **1.06** | **1.04-1.07^*^** | **1.03** | **1.02-1.05^*^** | **1.05** | **1.03-1.08^*^** |
| Pushing/pulling loads > 20kg | **1.54** | **1.13-2.09^*^** | 0.93 | 0.67-1.30 | 1.28 | 0.94-1.75 | 1.22 | 0.90-1.67 | **1.47** | **1.01-2.13^*^** |
| often work slightly bent posture | **2.13** | **1.46-3.11^*^** | **1.77** | **1.11 -2.81^*^** | **2.38** | **1.53-3.69^*^** | **2.34** | **1.52-3.59^*^** | **2.28** | **1.5-3.48^*^** |
| often work in heavily bent posture | **1.65** | **1.19-2.29^*^** | **1.85** | **1.32-2.60^*^** | **1.48** | **1.08-2.03^*^** | **1.46** | **1.07-2.01^*^** | 1.34 | 0.90-1.2 |
| slightly twisted posture for long | **1.71** | **1.23-2.37^*^** | 1.25 | 0.88-1.76 | 1.17 | 0.85-1.61 | **1.48** | **1.08-2.04^*^** | **2.07** | **1.35-3.17^*^** |
| Bent and twisted posture | **2.18** | **1.49-3.20^*^** | **1.93** | **1.33-2.79^*^** | **1.68** | **1.19-2.39^*^** | **1.45** | **1.02-2.05^*^** | **2.02** | **1.23-3.30^*^** |
| **Psychosocial factors** |  |  |  |  |  |  |  |  |  |  |
| Mental exhaustion | **1.73** | **1.27- 2.35^*^** | **2.18** | **1.54- 3.07^*^** | **1.86** | **1.36-2.54^*^** | **1.64** | **1.2-2.23^*^** | **2.22** | **1.51-3.27^*^** |
| Having part time jobs | 0.84 | 0.51-1.37 | **0.53** | **0.28-0.98^*^** | 0.84 | 0.5-1.40 | 1.02 | 0.62-1.68 | 0.93 | 0.51-1.69 |
| Supervision of others | **1.63** | **1.17-2.28^*^** | **2.46** | **1.61-3.75^*^** | **1.82** | **1.27-2.61^*^** | **2.33** | **1.61-3.36^*^** | **1.81** | **1.23-2.67^*^** |
